# A collective risk dilemma for tourism restrictions under the COVID-19 context

**DOI:** 10.1038/s41598-021-84604-z

**Published:** 2021-03-03

**Authors:** Manuel Chica, Juan M. Hernández, Jacques Bulchand-Gidumal

**Affiliations:** 1 Andalusian Research Institute DaSCI “Data Science and Computational Intelligence”, University of Granada, 18071 Granada, Spain; 2 School of Electrical Engineering and Computing, The University of Newcastle, Callaghan, NSW 2308 Australia; 3 Department of Quantitative Methods in Economics and Management, University of Las Palmas de Gran Canaria, Las Palmas, 35017 Spain; 4 TIDES Institute for Sustainable Tourism and Economic Development, University of Las Palmas de Gran Canaria, Las Palmas, 35017 Spain

**Keywords:** Computational science, Nonlinear phenomena

## Abstract

The current COVID-19 pandemic has impacted millions of people and the global economy. Tourism has been one the most affected economic sectors because of the mobility restrictions established by governments and uncoordinated actions from origin and destination regions. The coordination of restrictions and reopening policies could help control the spread of virus and enhance economies, but this is not an easy endeavor since touristic companies, citizens, and local governments have conflicting interests. We propose an evolutionary game model that reflects a collective risk dilemma behind these decisions. To this aim, we represent regions as players, organized in groups; and consider the perceived risk as a strict lock-down and null economic activity. The costs for regions when restricting their mobility are heterogeneous, given that the dependence on tourism of each region is diverse. Our analysis shows that, for both large populations and the EU NUTS2 case study, the existence of heterogeneous costs enhances global agreements. Furthermore, the decision on how to group regions to maximize the regions’ agreement of the population is a relevant issue for decision makers to consider. We find out that a layout of groups based on similar costs of cooperation boosts the regions’ agreements and avoid the risk of having a total lock-down and a negligible tourism activity. These findings can guide policy makers to facilitate agreements among regions to maximize the tourism recovery.

## Introduction

The pandemic of coronavirus disease 2019 (COVID-19) is one of the most shocking global crisis in the recent history. From Wuhan, China, to all over the world, COVID-19 has affected more than 100 million people and caused more than 2 million deaths as of January 31, 2021^1^. The effects of the pandemic originated by COVID-19 are not limited to the public health. Mobility restrictions and the halt of the economy have caused historical declines in every nation worldwide. In the EU, the GDP is forecast to decrease almost by 8% in 2020^2^, and most of the tourism-dependent countries have suffered a dramatic drop forecast in their GDP (e.g., 12% in Spain, 10% in Italy, and 9% in Greece, just to mention three of the most tourism-dependent economies in Europe)^3^.

A similar precedent to this COVID-19 pandemic is the 1918 Spanish Flu but in those days the world was not as connected as it is now. Since then, the travel and tourism sector has grown from being a marginal niche industry to one of the main industries worldwide. Notwithstanding tourism accounts for approximately 10% of the global GDP^4^, its contribution by country and region is far from homogeneous. Only in Europe there is a wide range of variation. Tourism accounts for less than 5% of the GDP in some countries (e.g., Poland) where, in other countries such as Croatia, the tourism contribution to GDP excesses 25%. Worldwide differences are even higher, with several countries where tourism accounts for more than half of their GDP^5,6^.

Many studies have been recently conducted to analyze the best policies and non-pharmaceutical interventions to contain the pandemic while reducing the economic damage^7–11^. Common conclusions are: the effectiveness of interventions depends on the local context such as timing^8^, they should be treated with caution with regard to policy-making decisions^9^, and must be focused on specific points of interest^11^. This myriad of policies have led to the fact that the majority of tourism destinations have implemented travel restrictions^12^ and have gone through different stages during the COVID-19 pandemic: from being able to accept tourists to have a complete lock-down and not being able to accept one single visitor. Consequently, finding the best conditions for the tourism sector under this COVID-19 context has gained attention in the tourism academic community^13,14^.

Additionally, researchers found that another key factor in controlling the COVID-19 pandemic is the role of coordination among regions and countries for the optimal performance of the proposed policies^10^. Private and public organizations must be coordinated to preserve pre-COVID-19 operational levels of the tourism and travel sector^15^. Tourism is an ecosystem that needs to bring together many actors from different geographic places^16^ and the tourist needs to be confident about the safety of her/his trip (i.e., a low perceived risk)^17^. Therefore, the coordination among restrictions and reopening between origin and destination tourism regions is nowadays necessary to control the epidemic and ameliorate the economic cost.

Our aim here is to analyze the conditions to achieve coordination among groups of regions or countries (we use the term “regions” from now) taking into account both the economic costs and the risk of a global lock-down because of a severe epidemiological situation. Some regions will implement restrictions on mobility and leisure activities to avoid the risk of extreme infection rates that would originate a global economic collapse. But consequently, these restrictions would lead to economic losses for those regions. Therefore, regions could be tempted not to cooperate to take advantage of the stable public health conditions which were achieved from the restrictions imposed by other regions.

Evolutionary game theory helps to represent the expected individual decisions to engage in economic costly restrictions in the COVID-19 pandemic context^18^. Specifically, we propose to frame the coordination problem above as a collective-risk dilemma (CRD)^19–21^. Collective risks arise when preserving common or public goods hinges on individual endowments and an investing failure hurts all the individuals of the population^22^. Specifically, CRDs are multiplayer public good games (PGGs) where every participant voluntarily contributes with part of her/his endowments to achieve a target, but they may suffer from a total loss with certain probability if the target is not achieved. The adoption of policies to prevent climate change by the global community has been the paradigmatic CRD game illustrated in the recent scientific literature^23^. The proposed CRD model will serve to analyze the conditions of cooperation (i.e., coordination on mobility restrictions and reopening policies) among different regions. As the level of tourism dependence in the region is heterogeneous, the economic loss derived from the implementation of these policies is heterogeneous as well. This heterogeneity is included in the evolutionary game by means of different contribution costs for the common good.

We analyze how this heterogeneity influences achieving a minimum level of cooperation. Specifically, we will answer to the question whether the heterogeneity of tourism-dependence among regions favors cooperation level. Additionally, the way regions are grouped to cooperate may also determine the global game outcome in terms of regions’ agreement. For example, groups can be formed at random without considering their tourism-dependent nature. Alternatively, groups can be formed according to similar tourism dependence (i.e., groups just having high dependent regions and groups having non-dependent regions). Therefore, a second question arises here: Is cooperation enhanced or detracted if groups are formed by regions having similar tourism-dependence?

In order to answer the above-mentioned questions we study the effects of adding heterogeneity to the cooperating costs of the players in the CRD. We define regions’ agreement (*RA*) as a cooperation indicator of the whole population of regions. We conduct a set of experiments to find the most beneficial group formation in terms of *RA* and global cooperation. For all the cases, the experiments evaluate different initial conditions, group sizes, and risk levels of a collective tourism and mobility shutdown. Finally, we apply the model to a case study based on the 312 regions of the EU NUTS2 classification. The regions at the NUTS2 level, that will be described in detail later in this manuscript, are a paradigmatic case of decentralized response to COVID-19. Although general EU policy recommendations have been recently laid down^24,25^, every country in the Union is autonomous to implement restrictions to free movement within its territory and with other countries or regions in the EU. The decentralized governance system in many countries (e.g. Spain, Italy, and Germany) makes a CRD model suitable to analyze coordination policies.

## Background

We address this problem of coordination for tourism in a COVID-19 context by proposing a collective risk dilemma (CRD) model. The CRD arises because the highest payoff of every participant is obtained by free-riding the contributions of other participants. Instead, if this strategy is followed by all players, the target would not be achieved and the risk of losing everything would be high.

The CRD has been formalized from the evolutionary game theory as a variation of public good game (PGG) where a threshold must be achieved to produce the public good^26^. In addition, a certain risk of losing a big part of the remaining endowment of every participant is assumed in case of the threshold is not achieved^20,21,27^. Several general findings in this kind of games have been revealed in previous studies. For example, Wang et al. find that increasing risk levels of big loss favors cooperation^20^. In the CRD models, players are organized in groups of a certain size and the game is restricted to every group. In general, the studies show that the group size works against cooperation in the sense that achieving threshold is more difficult for large groups^21^. Larger groups are harder to coordinate because of their overall lower success frequencies^22^. These general results are found in the subsequent CRD models proposed in the last decade.

Other contributions extend previous models by including the role of punishment institutions. Pacheco et al. find that local institutions (those restricted to participants in every group) are more efficient to promote cooperation than global institutions^23^. Following this line, the optimal combination of punishment and reward policies was analyzed in^28^, whereas the effect of different tax and fine strategies was studied by Couto et al.^29^. The latter contribution finds that a graduated punishment/tax strategy enhances global cooperation rates more than having a fixed amount. Other interesting factors analyzed in the previous literature are the role of migration^30^ and the timing of the contribution of every participant^31^. Recently, the classical CRD model framework has been extended by defining two environmental states (prosperous and degraded) which evolve and interact to each other^32^. The general results agree with previous findings and extend them by analyzing the effect of time preferences and magnitude of collapse. Finally, behavioral studies showed that the best mechanisms to avoid collective risks depend on an interaction between behavioral type, communication, and timing^22^.

Previous contributions have analyzed the role of heterogeneity in CRD models from different points of view. For example, Santos et al. analyze how cooperation arises in PGG with heterogeneous social network structures^33^. Results point that cooperation is favored in scale-free structures with respect to homogeneous regular graphs. As it is the case in this paper, several previous contributions assume different types of participants in the CRD. Specifically, Wang et al. include the effect of wealth inequality in *n* participants in games with infinite population and a fixed contribution per participant^34^ . The main conclusion is that rich contributors con sustain cooperation level above threshold. Abou Chakra and Traulsen assume two different types of contributors (rich and poor) in a CRD model with two rounds, where the rich ones can contribute with two different positive amounts every round^35^. The results show a strategic behavior of poor contributors who mostly cooperate once the rich contributors have contributed in the first round. Vasconcelos et al. also propose a model with two types of participants (rich and poor) with dissimilar contributions^36^. The main result is that wealth inequality enhances global cooperation if players revise strategies from comparison with any other player, but this is not the case if players only take into account players belonging to her/his own type when revising strategies. The impact on how groups are formed in a game with heterogeneous players has not been analyzed in the CRD relevant literature yet.

## Methods

### Model definition and groups of regions

The proposed CRD model is formed by a finite set of *Z* players which represent the regions that make decisions about adopting or not public health recommendations. Each player *i* chooses, at every time step *t*, a strategy *s* from two possibilities ($s(i)=\{C,D\}$): being a cooperator, which means assuming the restrictions and public health recommendations (*C*), or being a defector and ignore those recommendations (*D*). Thus, regions adopting a cooperation strategy *C* will suffer from a direct drop in their income from tourism and free mobility conditions.

The model also includes, as in previous CRDs, a risk parameter $r \in [0,1]$ for a global disaster. This risk means falling in a global lock-down for the members of the group because of the high incidence of the COVID-19 for the regions of the group. Note again that the goal of the proposed model is not the virus contagion modeling and the collective risk is a critical epidemiological situation for the regions and therefore, an economic collapse for the regions involved. This risk value *r* also measures the difficulty of the social dilemma. Lower values of *r* correspond to less perceived risk for the public health and economic collapse and therefore, cooperation becomes more difficult.

Players of the population make agreements and cooperate in invariable groups of size *N* which represent groups of regions (i.e., inside a country or within an international alliances or international work-groups). By default in CRDs, these groups are formed at random^36^ but we will investigate the role and heterogeneity of the groups’ formation in the experiments of this work. A group of regions or players is successful when its number of cooperators achieves a minimum threshold, defined by a ratio $m \in [0,1]$. The number of cooperators in a group *k*, denoted by $C^k$, must be equal or greater than *mN* for a group of regions to be successful and obtain an agreement that implement the COVID-19 restrictions to prevent the economic breakdown. We call this achievement, for all the formed groups, as regions’ agreement ($RA \in [0, 1]$). *RA* is the ratio of groups which obtain the required number of cooperators and will be the performance cooperation indicator for the model during our experimental analysis.

When a region cooperates and restricts mobility, tourism activity critically stops and the income losses from these restrictions occur in the region. These losses or costs for a cooperating region *i* are denoted by $c_i \in [0,1]$. The dependence of regions to tourism is heterogeneous and thus, this cost $c_i$ in our evolutionary model is higher for tourism dependent regions. If a region is fully dependent on tourism, its income will be zero when the region adopts the public health restrictions and thus, its cost $c_i$ is set to 1 (i.e., its initial tourism endowment is totally lost). When a region *i* is not cooperating to the common good to stop the spread of the disease (i.e., a *free rider* with strategy *D*), its cost is set to 0.

The payoff $w_i$ of a region or player *i* depends on its strategy *s*(*i*) at the last time step within its group. If $s(i) = D$, then the payoff of region *i*, belonging to a group *k*, is given by $w_i = \Pi _D$ (see Equation 1). *N* stands for the size of the group, $C^k$ is the number of the cooperators in the group *k*, and *m* is the minimum ratio of cooperators in the group to achieve the regions’ agreement. $\Theta (x)$ is a Heaviside function returning 1 when $x \ge 0$, 0 otherwise. If the region is cooperating for its group *k* (i.e., $s(i) = C$), then the region pays a cost $c_i$ for adopting the public health restrictions. The payoff of a cooperating region *i* is $w_i = \Pi _C = \Pi _D - c_i$. As previously stated, the cost $c_i$ is different for each player as it depends on the tourism dependency of each region.

1 $$\begin{aligned} \Pi _D = \Theta ( C^k - mN ) + (1-r) [1 - \Theta ( C^k - mN )]. \end{aligned}$$

### Evolutionary dynamics

After playing the game and calculating their payoffs at every time step *t*, the regions or players can update their strategies according to the payoffs received from its invariable group. Regions (players) then decide which strategy to choose based on the strategies followed by other regions of the population in the previous time step (i.e., $t - 1$) and their own payoff^37^. The strategy update follows an evolutionary procedure based on the imitation in a well-mixed population. It means a given region or player *i* can imitate any other region from the population, even from other groups, but this region *i* plays the coordination game with the same group of regions for all the time steps of the simulation. This process can be seen as a social learning process^38^ to build the collective behavior of the population^39^.

We have considered the Fermi function as the evolutionary update rule, which is applied synchronously. The Fermi rule is a stochastic pairwise comparison rule, where regions or players can make mistakes during the imitation process. This means that a region can copy the strategy of another region having a worse payoff. Mathematically, a region *i* having strategy *X* adopts strategy *Y* of region *j* (randomly selected from the population) with a probability given by Equation 2. $\beta$ is the intensity of selection parameter, set to 0.5 in all the experiments of this work. Additionally, players can also change their strategies by adopting a strategy at random, following a mutation mechanism with probability $\mu$ equals to 0.01, as done in other CRDs^36,40^.

2 $$\begin{aligned} prob_i(Y) = \frac{1}{1 + e^{- \beta (w^{t-1}_j - w^{t-1}_i)}}. \end{aligned}$$

### EU NUTS2 real data and model setup

**Figure 1:**
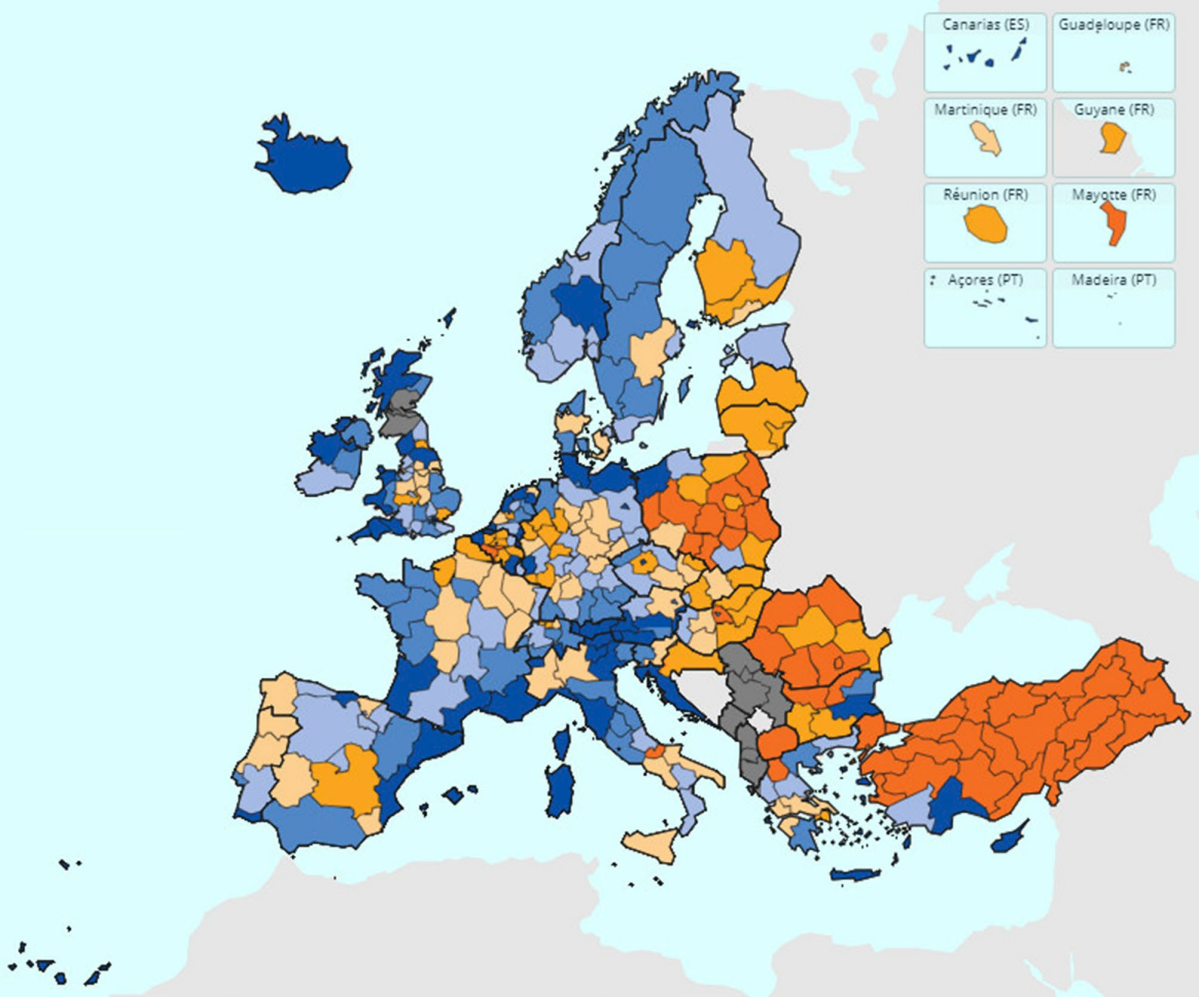
Map showing the EU NUTS2 regions colored by their tourism dependence. Regions in blue are those with the highest tourism dependence while regions in orange have the lowest tourism dependence. If tourism information is not available, the region is colored in gray. Source: Eurostat.

We feed the heterogeneous costs of the regions of the model by taking into account the real context of the EU regions. We use the EU NUTS2 classification^41^, defined as “basic regions for the application of regional policies” which is precisely the interest of this research. This classification includes 312 administrative regions in Europe (see Fig. 1 where the reader can see these regions within their respective countries). Most of the well-known tourist regions in Europe are defined through this NUTS2 classification (e.g., Canary Islands, Sicily, Tuscany, Algarve and Tirol, among others). In many European countries, these NUTS2 regions are responsible for their health and tourism policies. In fact, EU countries such as Germany, Spain, Belgium, and Italy are among the five most decentralized countries in the world, together with USA^42^.

We have collected data from the EU NUTS2 classification with respect to nights spent at tourist accommodation establishments per inhabitant. This data is used to set the $c_i$ values of the model as these values represent a measure of the economic costs when a lock-down and/or restrictions are applied for tourism-dependent regions. As can be seen in the map of Fig. 1, regions have a diverse tourism-dependence for all the countries (groups). We have generated two clusters of regions by taking into account the number of night stays per inhabitant for each region. We set a cut-off value of 4 ($\alpha =0.04$), meaning that regions with a ratio of more than 4 night stays per inhabitant were considered as tourism-dependent while those with a ratio of less than 4 were considered non-dependent. The cut-off value of 4 was set since it splits the regions in two clusters: 20% of dependent having more than 4 stays per inhabitant and 80% of non-dependent with less than 4 stays. The cluster of tourism-dependent regions has an averaged cost of $c^T = 0.158$. In contrast, the cluster of non tourism-dependent regions has an averaged cost of $c^{NT} = 0.013$. The averaged cost value of all the regions of the data is ${\hat{c}}=0.04$.

**Figure 2:**
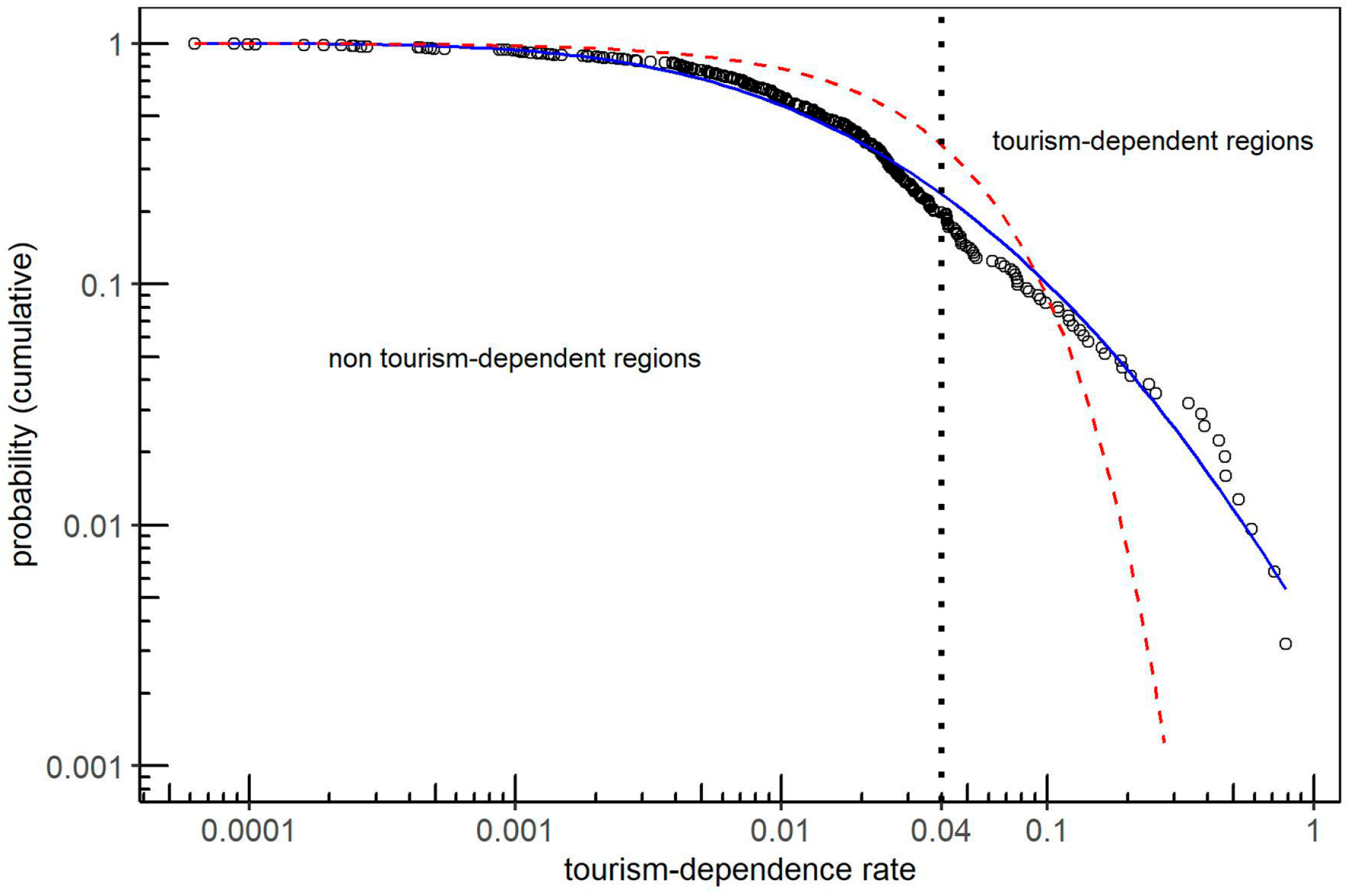
Cumulative distribution of the real costs for the 312 regions of the EU NUTS2 (circles). Blue solid line represent the fitted log-normal distribution and red dashed line the fitted exponential distribution. The vertical line $\alpha =0.04$ divides the regions in two clusters: non tourism-dependent and tourism-dependent.

An exponential and log-normal distributions are fitted to the real costs data of the NUTS2 regions. Figure 2 shows the histogram of the real costs data *c*, a vertical dotted line for the $\alpha =0.04$ cut-off to generate two clusters of tourism dependent and non-dependent regions, and the two fitted distributions. The first is a fitted exponential distribution $c \sim Exp(\lambda )$, shown in the figure as a red dashed line, with $\lambda = 24.30$. The second is a fitted log-normal distribution $\ln (c) \sim {\mathcal {N}}(\mu , \sigma ^2)$, shown in the figure as a blue solid line, with parameters $\mu = -4.39$ and $\sigma = 1.63$.

For the experimental study we run the model for 30 independent Monte-Carlo (MC) realizations and $Z=3,000$ players to have robust results but when applying the model to the real NUTS2 case study we use $Z=312$. The model is fed with heterogeneous costs from the fitted distributions. When using two clusters of regions, tourism dependent and non-dependent, having costs $c^T = 0.158$ for 20% of the regions and $c^{NT} = 0.013$ for the remaining 80% for them. Finally, we use a homogeneous cost of ${\hat{c}}=c_i=0.04, \forall i$, which is the averaged cost of all the regions of the distribution.

**Figure 3:**
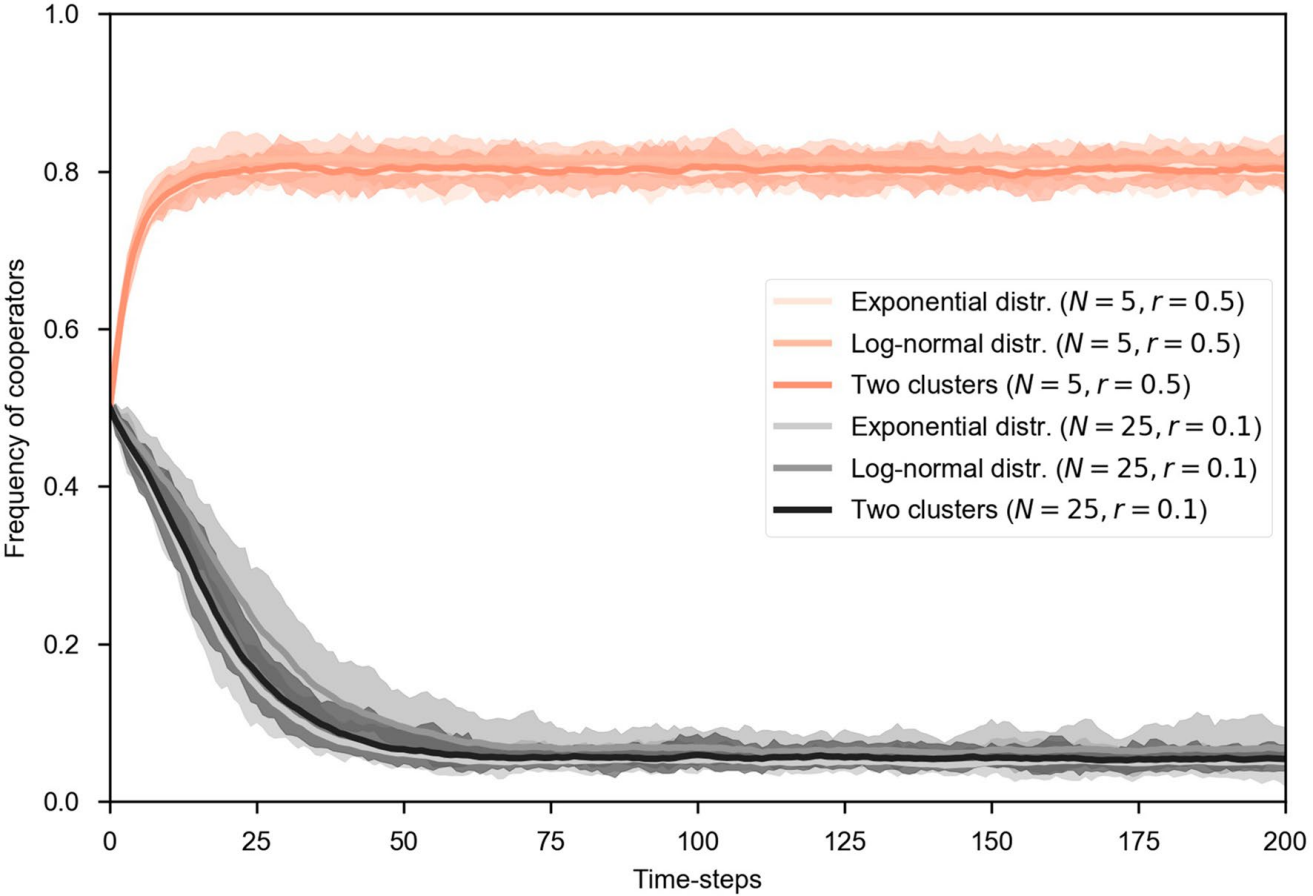
Evolution of the tourism dilemma using heterogeneous costs for the regions (players) for different risk *r* values and group sizes for 200 time steps. The solid line is the average of the MC runs and the semi-transparent region denotes the maximum and minimum values obtained in these 30 MC runs.

The simulation results were obtained by averaging the last 25% of the simulation time steps in the independent MC runs. Finally, each model is run for 200 time steps, where all the realizations reach a stationary stable state and deviation from the MC realizations is low, as seen in Fig. 3. In this plot we see the evolution of the model for $Z=3,000$ players, $m=0.5$, groups assigned at random of size $N=5$ and $r=0.5$ and $N=25$ with $r=0.1$. We see how stationary state is reached easily for all the instances of models having heterogeneous costs generated by exponential and log-normal distributions and two clusters of regions (i.e., tourism dependent and non-dependent).

## Analysis of the results

The analysis starts by comparing the dynamics’ difference and levels of cooperation when using heterogeneous costs with respect to two clusters of regions and homogeneous costs. Later, the analysis focuses on the groups formation where we compare the impact of grouping regions with similar costs on cooperation. Finally, we show the application of the model to the real case of 312 NUTS2 regions with their real heterogeneous costs.

### Impact of considering heterogeneity in the regions’ tourism costs

The goal here is to see cooperation differences when introducing heterogeneous costs for regions when applying public health restrictions for the common good of their groups. Then, we compare three different model specifications for costs while maintaining the rest of the model’s parameters. First, we run the model with heterogeneous costs $c_i$ following a log-normal distribution, fitted to the real NUTS2 regions’ data. Second, we use the two clusters of regions with two costs values, distinguishing them by tourism dependent and non-dependent. Third, we assume that all the regions are cooperating, and that the cost for all of them is an average global cost. For all the scenarios, the groups formation is done at random and for three group sizes *N*.

**Figure 4:**
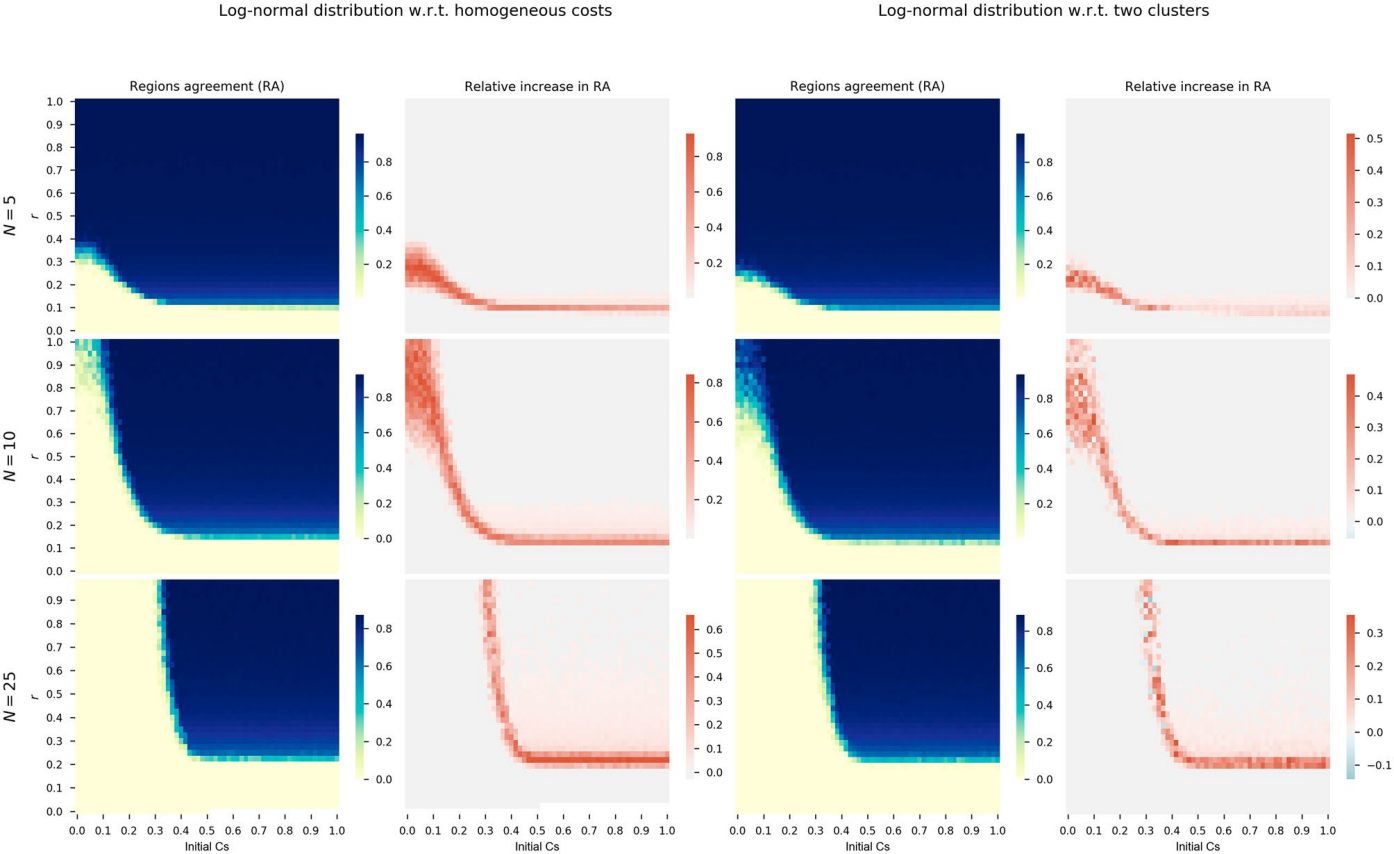
First and third columns show heatmaps of absolute *RA* when considering equal global costs and two groups of regions, respectively. The second column shows relative *RA* differences when using log-normal distribution costs with respect to equal costs for all the regions. Fourth column shows heatmaps with relative *RA* differences of log-normal distribution costs with respect to two clusters of regions. Each row of the panel shows results for different group sizes (*N*) and $m=0.5$.

Figure 4 shows a panel of pairs of heatmaps with a double comparison. Each pair of heatmaps has a first one showing the regions’ agreement (*RA*) of the heterogeneous version of the model and a second one, ranging from white to red colors, with a relative increase with respect to the second and third scenario. The first two columns compare the heterogeneous costs generated by a log-normal distribution fitted to the real NUTS2 tourism data with respect to having the same cost for all the regions (an averaged cost of ${\hat{c}} = 0.04$). The third and fourth columns of the panel show the comparison between the generated log-normal distribution costs with respect to two groups of regions (20% of tourism dependents with a cost of $c^T = 0.158$ and 80% of non-dependents with a cost of $c^{NT} = 0.013$). The heatmaps are built from a sensitivity analysis on the initial number of cooperators (x-axis) and risk parameter *r* (y-axis). Finally, we also investigate three different groups sizes ($N=5$ in the first row, $N=10$ in the second row, and $N=25$ in the third row).

First and third columns show heatmaps of absolute regions’ agreement (*RA*) when considering equal global costs and two groups of regions, respectively. The second column shows relative *RA* differences when using log-normal distribution costs with respect to equal costs for all the regions. Fourth column shows heatmaps with relative *RA* differences of log-normal distribution costs with respect to two groups of regions.

We observe from the heatmaps that results are not equivalent when considering homogeneous, two-clusters, and heterogeneous costs. Even if the homogeneous and two-clusters versions of the model come from averaging the real costs, differences are clear. For all the group sizes, initial cooperation conditions, and level of risk, cooperation and therefore, *RA* is clearly higher when considering heterogeneous costs. The *RA* obtained with heterogeneous costs is at least 30% higher than when using equal costs for all the regions in the transition phase from null cooperation (light yellow areas) to high cooperation (blue areas of the heatmaps). There is also a *RA* increase in the high cooperation area although of a lower value.

In the case of comparing against two differentiated groups of regions with two different cooperation costs, the increase in *RA* is also significant if we introduce a heterogeneous costs distribution. Again, the results are robust for all the group sizes, initial conditions, and levels of risk. The higher increase is observed in the transition area although there is a less significant increase beyond this transition and towards the cooperation zone (i.e., high number of initial cooperators and high risk values).

Different group sizes does not affect the increase in the *RA* for the heterogeneous version of the model which is invariant for all the *N* values. However, we see how increasing the group of players or regions is more difficult for promoting cooperation. In view of the results of Fig. 4, there is a shift of the transition area from null to high cooperation when increasing the group size. This transition phase is also thinner when increasing the group size (compare for instance $N=5$ and $N=25$ in the relative increase heatmaps). This behavior is expected from previous similar CRD studies^36^.

**Figure 5:**
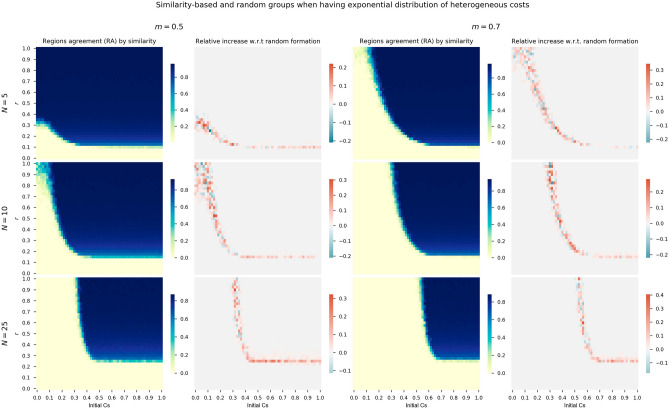
First and third columns show heatmaps of absolute *RA* when considering exponential distribution costs and grouping regions at random for thresholds $m=0.5$ and $m=0.7$. The second and fourth columns show relative *RA* differences when using exponential distribution costs and grouping by similarity with respect to random groups.

### Group formation by similarity and at random

In this section we inspect an important decision when organizing regions in groups for increasing their cooperation and agreement. By default in CRDs, regions or players are homogeneous and they are normally assigned to groups at random, as also done in the previous section. However, given the heterogeneity of the regions or players in terms of cooperating costs because of their different dependence to tourism, this set of experiments evaluates if grouping regions by similar cooperation costs $c_i$ increases cooperation and *RA*.

**Figure 6:**
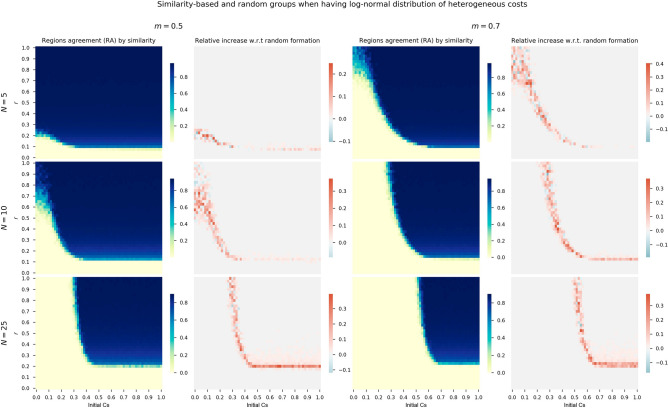
First and third columns show heatmaps of absolute *RA* when considering log-normal distribution costs and grouping regions at random for thresholds $m=0.5$ and $m=0.7$. The second and fourth columns show relative *RA* differences when using log-normal distribution costs and grouping by similarity with respect to random groups.

**Figure 7:**
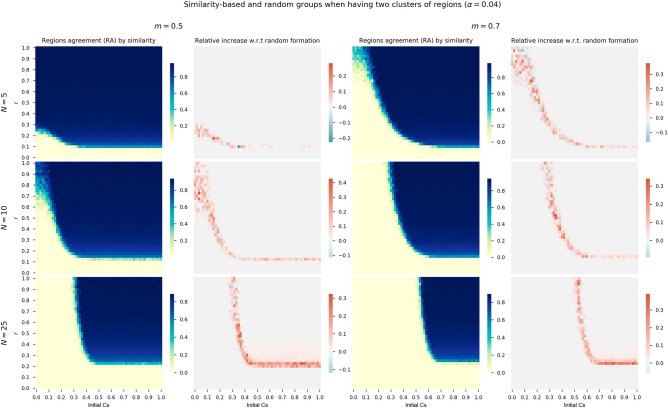
First and third columns show heatmaps of absolute *RA* when considering two clusters of regions ($\alpha =0.04$) and grouping regions at random for thresholds $m=0.5$ and $m=0.7$. The second and fourth columns show relative *RA* differences when using two clusters of regions grouped by similarity with respect to random groups.

In order to answer this question we launch sensitivity analysis for the initial cooperation conditions and risk levels for different group sizes and minimum threshold for agreement *m*, similarly to the experiments of the previous section. We compare the *RA* relative increase of grouping regions with similar costs (i.e., tourism dependence) with respect to a grouping at random. This analysis is performed for costs generated by the fitted exponential distribution in panel of Fig. 5, log-normal distribution in panel of Fig. 6, and also for the two clusters of regions configuration in the panel of Fig. 7.

The panel of heatmaps for the exponential distribution of Fig. 5 and log-normal distribution of Fig. 6 show similar behaviors. First, the levels of *RA* of the heatmaps of the first and third columns are very similar between both distributions. As observed in the previous section, the cooperation area in blue shrinks as the group size *N* becomes higher. Similar behaviors are also observed for $m=0.5$ (first group of two columns) and $m=0.7$ (second group of two columns) for both distributions, with narrower cooperation areas (heatmaps’ cells in blue) when the minimum ratio *m* is 0.7.

If we focus the analysis on the second and fourth columns of the both panels, where the relative increase with respect to random formation is depicted, we see how there is an increase in cooperation when regions are assigned to groups based on similarity in terms of costs. Although there is some variability and negative increases, mainly with high risk levels, the general picture is of a *RA* increase.

We also performed the same analysis but for the two clusters configuration to compare its results with the continuous distributions of costs. Panel of Fig. 7 shows the same matrix of heatmaps than for the exponential and log-normal distributions. The results are also similar to the previous cases. There is an increase in RA levels when grouping regions by similar costs with respect to a random assignment. Better cooperation results apply to the three studied group sizes *N* (each row of the panel), for the two *m* values, and for the whole range of initial cooperators and risk levels. Therefore, we can conclude that the benefits of disposing regions or players in this CRD by similarity in their groups facilitate the *RA* both with heterogeneous real costs and two clusters of tourism dependent and non-dependent.

### Application to the real EU NUTS2 case

**Figure 8:**
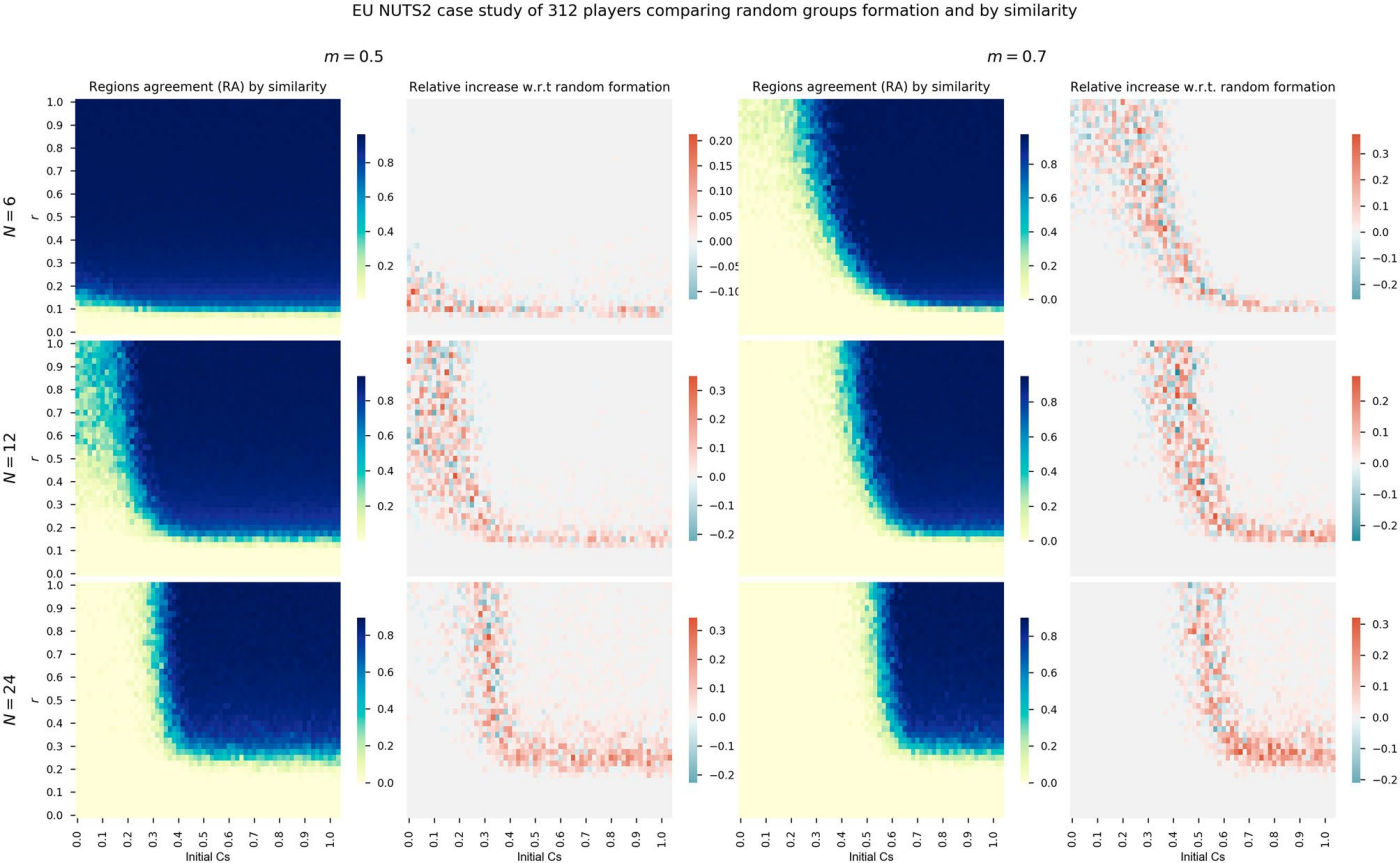
First and third columns show heatmaps of absolute *RA* when considering the real data from the 312 NUTS2 regions, grouped at random for thresholds $m=0.5$ and $m=0.7$. The second and fourth columns show relative *RA* differences when grouping by similarity with respect to random groups.

In this last section of the experimental study, the direct NUTS2 regions of the EU are used to feed the model. The configuration implies having $Z=312$ players with groups of $N=6$, $N=12$, and $N=24$, and two minimum threshold of cooperation, *m* for 50% of the members and 70% of them. The costs $c_i$ for the 312 players are directly obtained from the NUTS2 tourist night stays per inhabitant data, from 0 to 1. Panel of Fig. 8 shows the heatmaps for the real case study comparing the grouping by similarity and its relative increase with respect to a random formation. Two first columns are for $m=0.5$ while the last two columns correspond to $m=0.7$ Each row of the panel corresponds to a group size (*N*). As done in the previous section, we evaluate all the possible initial fraction of cooperators and risk levels defined by the *r* parameter.

Similar trends to the ones observed for the case of 3000 players can be seen although the variability both in the relative increase and RA of the similarity groups configuration is higher. In the lower part of the transition phase of the heatmaps, where *r* is low, the relative increase is always positive. When the *r* values increase, the variability is higher. In the blue area of the heatmaps, where *RA* is high, the formation by similarity also obtains a regular positive increase, although with lower values. To sum up, this real case study confirms that grouping by similarity achieves higher *RA* and cooperation, despite the higher variability given by the lower number of regions.

## Final discussion

A convenient management of lock-downs and mobility restrictions of the countries and regions in a COVID-19 context is crucial for both public health and affected economic sectors such as tourism. Therefore, it is important for regions to cooperate for the common good to achieve an agreement. This is the reason why we proposed here an evolutionary game model based on a well-known subset of PGGs, namely the collective risk dilemma (CRD), to model the affected regions’ cooperation to avoid the collective economic collapse. This collapse comes from an uncontrolled virus spread that would end in strict confinements and negligible tourist income. The presented model is the first to consider the CRD framework for the economic problem of tourism restrictions because of the COVID-19 pandemic. EU NUTS2 regions are used as an inspiration for the modeling and their data is used to enrich the study.

The regions are the players of the CRD and are grouped for obtaining regions’ agreements when the number of cooperators achieve a minimum threshold. Our proposed evolutionary game theory model employs a cost for regions when they cooperate because of their mobility and tourism restrictions. This cost is directly linked to their tourism dependence and, given their heterogeneity among the regions, these costs are heterogeneous in the model. The regions or players in the model follow an evolutionary update rule to change their strategy based on the payoffs obtained in their groups.

The experiments were first focused on observing how heterogeneity in the costs for cooperating regions increase the cooperation, and therefore the regions’ agreement (*RA*) for all the evaluated conditions. Based on the EU NUTS2 real data, we consider three ways of cost heterogeneity: a) two clusters of regions with equal costs, b) a log-normal continuous distribution of costs, and c) an exponential distribution of costs. The use of continuous distributions of heterogeneous costs makes the analytical treatment of the model impractical. On the contrary, the agent-based simulation allows studying the model outcomes for a large spectrum of initial conditions and values of parameters. As expected, major differences in the *RA* by assuming heterogeneous costs are observed in the transition phase of the initial conditions between two extreme stable cooperation values, although there is also a cooperation increase for high levels of final cooperation. The simplification of using two different clusters of regions reduces the final cooperation with respect to having a continuous distribution of heterogeneous costs (both exponential and log-normal). Previous works considered different groups of players^36^ but our results show that, in terms of costs, a complete heterogeneity in the cooperation costs are valuable and also facilitates the final cooperation. Nevertheless, our results confirm previous findings such as a positive effect on cooperation by increasing risk levels of collective failure and low number of players in the groups. As done in previous CRD models^21,34,36^, the final stable cooperation levels depend on the initial cooperation of the players and the most frequent final cooperation rates are either null or high.

Second and more importantly for decision makers, we studied whether the initial formation of the groups of regions affects the cooperation and *RA*. We conducted a set of experiments using the above-mentioned settings of heterogeneous costs for different initial conditions, risk levels, groups sizes, and minimum thresholds for group agreement. The application of the model to a more reduced set of players to mimic the real case of NUTS2 312 regions was also tested in our experimental study. The variability of the results is higher than in the synthetic experiment but their dynamics are equivalent. Differences in the results with respect to the experimental case with a larger population (i.e., 3, 000 players) points to the effect of low and finite population in the cooperation levels, as it was shown in previous analytical studies^27^.

The conclusion is that a group formation by similarity (i.e., regions with similar tourism-dependence are placed in the same groups) enhances the cooperation among the regions and our main cooperation indicator, *RA*, if we compare it with a traditional random formation. Again, the most significant differences in *RAs* are observed in the transition phase of the initial conditions between two extreme stable cooperation values. This final conclusion of the present research can be helpful for policy makers. Although the European Union was formed almost 30 years ago, there is still a trend for each country to consider itself relatively independent when it comes to policy making. However, in a global and challenging crisis like the current one, our results show that all regions will much better off by cooperating. Surprisingly enough, the best case scenario of this cooperation takes place between regions that have a similar dependence on tourism. For instance, high tourism-dependent regions (e.g., Canary Islands, Ionian Islands, Sicily, Algarve, Tirol) should cooperate together even if they belong to different countries and sometimes have a direct competition in attracting tourists.

This work represents the first CRD for modeling lock-downs and mobility restrictions under the COVID-19 context *albeit* presents some limitations. The model does not include side-effects for defecting regions when their flow of tourists are reduced because of the restrictions in the regions from which they receive tourists. Even if a tourism-dependent region does not follow any restrictions and is open to tourism, the drop in their touristic numbers will be significant because other regions have a limited mobility. Spatial information was not included in the model but it could enrich it by adding distances and/or in-out touristic flows among regions. Future works can include social networks or spatial lattices to take into account the latter effects. Additionally, punishment and reward policies can be injected into the model to see the effects on the final cooperation levels. And finally, analytical studies of a more simplified version of the model can also help towards the mathematical comprehension of the obtained outcomes and dynamics.

## References

[1] Worldometers. Covid-19 coronavirus pandemic. Accessed 21 January 2021. https://www.worldometers.info/coronavirus/ (2021).

[2] European Commission. Autumn 2020 economic forecast: rebound interrupted as resurgence of pandemic deepens uncertainty. Accessed 21 January 2021. https://ec.europa.eu/commission/presscorner/detail/en/ip_20_2021 (2020).

[3] European Commission. Economic performance by country. Accessed 21 January 2021. https://ec.europa.eu/info/business-economy-euro/economic-performance-and-forecasts/economic-performance-country_en (n.d.).

[4] World Economic Forum. The travel & tourism competitiveness index 2019. Accessed 21 January 2021. http://reports.weforum.org/travel-and-tourism-competitiveness-report-2019/files/2019/09/OVERALL-RESULTS.pdf (2019).

[5] Council, W.T. Economic impact reports. Accessed 21 January 2021. https://wttc.org/Research/Economic-Impact (2020).

[6] Fernandes, N. Economic effects of coronavirus outbreak (covid-19) on the world economy. SSRN 3557504 (2020).

[7] Padhi A (2020). Studying the effect of lockdown using epidemiological modelling of covid-19 and a quantum computational approach using the Ising spin interaction. Sci. Rep..

[8] Haug N (2020). Ranking the effectiveness of worldwide covid-19 government interventions. Nat. Hum. Behav..

[9] Soltesz K (2020). The effect of interventions on covid-19. Nature.

[10] Svoboda, J., Tkadlec, J., Pavlogiannis, A., Chatterjee, K. & Nowak, M. A. Infection dynamics of covid-19 virus under lockdown and reopening. arXiv preprint 2012.15155 (2020). 10.1038/s41598-022-05333-5 PMC8795434 35087091

[11] Chang S (2020). Mobility network models of covid-19 explain inequities and inform reopening. Nature.

[12] UNWTO. Covid-19 travel restrictions. Accessed 21 January 2021. https://www.unwto.org/news/covid-19-travel-restrictions (2020).

[13] Qiu RT, Park J, Li S, Song H (2020). Social costs of tourism during the covid-19 pandemic. Ann. Tour. Res..

[14] Altuntas F, Gok MS (2020). The effect of covid-19 pandemic on domestic tourism: a dematel method analysis on quarantine decisions. Int. J. Hosp. Manag..

[15] Škare M, Soriano DR, Porada-Rochoń M (2020). Impact of covid-19 on the travel and tourism industry. Technol. Forecast. Soc. Change.

[16] Leiper N (1979). The framework of tourism: towards a definition of tourism, tourist, and the tourist industry. Ann. Tour. Res..

[17] Lepp A, Gibson H (2003). Tourist roles, perceived risk and international tourism. Ann. Tour. Res..

[18] Aubrecht P, Essink J, Kovac M, Vandenberghe A-S (2020). Centralized and decentralized responses to COVID-19 in federal systems: US and EU comparisons. SSRN Electron. J..

[19] Milinski M, Sommerfeld RD, Krambeck HJ, Reed FA, Marotzke J (2008). The collective-risk social dilemma and the prevention of simulated dangerous climate change. Proc. Natl. Acad. Sci. USA.

[20] Wang J, Fu F, Wu T, Wang L (2009). Emergence of social cooperation in threshold public goods games with collective risk. Phys. Rev. E - Stat. Nonlinear Soft Matter Phys..

[21] Santos FC, Pacheco JM (2011). Risk of collective failure provides an escape from the tragedy of the commons. Proc. Natl. Acad. Sci. USA.

[22] Wang Z (2020). Communicating sentiment and outlook reverses inaction against collective risks. Proc. Natl. Acad. Sci..

[23] Pacheco JM, Vasconcelos VV, Santos FC (2014). Climate change governance, cooperation and self-organization. Phys. Life Rev..

[24] Union, E. Council recommendation on a coordinated approach to the restriction of free movement in response to the COVID-19 pandemic (press release, 13 October 2020) (2020).

[25] Union, E. Council Recommendation amending Council Recommendation (EU) 2020/1475 of 13 October 2020 on a coordinated approach to the restriction of free movement in response to the COVID-19 pandemic (2021).

[26] Pacheco JM, Santos FC, Souza MO, Skyrms B (2009). Evolutionary dynamics of collective action in N-person stag hunt dilemmas. Proc. R. Soc. B: Biol. Sci..

[27] Santos FC, Vasconcelos VV, Santos MD, Neves PN, Pacheco JM (2012). Evolutionary dynamics of climate change under collective-risk dilemmas. Math. Model. Methods Appl. Sci..

[28] Góis AR, Santos FP, Pacheco JM, Santos FC (2019). Reward and punishment in climate change dilemmas. Sci. Rep..

[29] Couto MC, Pacheco JM, Santos FC (2020). Governance of risky public goods under graduated punishment. J. Theor. Biol..

[30] Chen X, Szolnoki A, Perc M (2012). Risk-driven migration and the collective-risk social dilemma. Phys. Rev. E - Stat. Nonlinear Soft Matter Phys..

[31] Abou Chakra M, Traulsen A (2012). Evolutionary dynamics of strategic behavior in a collective-risk dilemma. PLoS Comput. Biol..

[32] Barfuss W, Donges JF, Vasconcelos VV, Kurths J, Levin SA (2020). Caring for the future can turn tragedy into comedy for long-term collective action under risk of collapse. Proc. Natl. Acad. Sci. USA.

[33] Santos FC, Santos MD, Pacheco JM (2008). Social diversity promotes the emergence of cooperation in public goods games. Nat..

[34] Wang J, Fu F, Wang L (2010). Effects of heterogeneous wealth distribution on public cooperation with collective risk. Phys. Rev. E - Stat. Nonlinear Soft Matter Phys..

[35] Abou Chakra M, Traulsen A (2014). Under high stakes and uncertainty the rich should lend the poor a helping hand. J. Theor. Biol..

[36] Vasconcelos VV, Santos FC, Pacheco JM, Levin SA (2014). Climate policies under wealth inequality. Proc. Natl. Acad. Sci..

[37] Chica M, Chiong R, Ramasco JJ, Abbass H (2019). Effects of update rules on networked n-player trust game dynamics. Commun. Nonlinear Sci. Numer. Simul..

[38] Nowak MA, Tarnita CE, Antal T (2010). Evolutionary dynamics in structured populations. Philos. Trans. R. Soc. Lond. B: Biol. Sci..

[39] Perc M (2019). The social physics collective. Sci. Rep..

[40] Vasconcelos VV, Santos FC, Pacheco JM (2013). A bottom-up institutional approach to cooperative governance of risky commons. Nat. Clim. Change.

[41] Eurostat. Nights spent at tourist accommodation establishments by nuts 2 regions. Accessed 21 January 2021. https://ec.europa.eu/eurostat/web/products-datasets/-/tgs00111 (2020).

[42] Schakel, A. H., Danailova, A., Gein, I. & Hegewald, S. Final report on updating the regional authority index (rai) for 45 countries (2010–2016) (2018).

